# A cluster-based approach to selecting representative stimuli from the International Affective Picture System (IAPS) database

**DOI:** 10.3758/s13428-016-0750-0

**Published:** 2016-06-10

**Authors:** Alexandra C. Constantinescu, Maria Wolters, Adam Moore, Sarah E. MacPherson

**Affiliations:** 1 Department of Psychology, School of Philosophy, Psychology and Language Sciences, University of Edinburgh, 7 George Square, Edinburgh, EH8 9JZ UK; 2 Centre for Cognitive Ageing and Cognitive Epidemiology, University of Edinburgh, Edinburgh, UK

**Keywords:** IAPS, International affective picture system, Cluster analysis, Stimulus selection, Emotion

## Abstract

The International Affective Picture System (IAPS; Lang, Bradley, & Cuthbert, 2008) is a stimulus database that is frequently used to investigate various aspects of emotional processing. Despite its extensive use, selecting IAPS stimuli for a research project is not usually done according to an established strategy, but rather is tailored to individual studies. Here we propose a standard, replicable method for stimulus selection based on cluster analysis, which re-creates the group structure that is most likely to have produced the valence arousal, and dominance norms associated with the IAPS images. Our method includes screening the database for outliers, identifying a suitable clustering solution, and then extracting the desired number of stimuli on the basis of their level of certainty of belonging to the cluster they were assigned to. Our method preserves statistical power in studies by maximizing the likelihood that the stimuli belong to the cluster structure fitted to them, and by filtering stimuli according to their certainty of cluster membership. In addition, although our cluster-based method is illustrated using the IAPS, it can be extended to other stimulus databases.

**Electronic supplementary material:**

The online version of this article (doi:10.3758/s13428-016-0750-0) contains supplementary material, which is available to authorized users.

## Introduction

It is now widely accepted that emotion plays a critical role in human psychology and is inextricably entwined with behavior and cognition. Yet, a major challenge that emotion researchers face is conceptualizing the relationship between various kinds of emotions and mapping their collective impact on other psychological processes (e.g., Ito, Cacioppo, & Lang, 1998; Lane et al., 1997; LeDoux, 1996). Perhaps the most widely used tool in this pursuit is the International Affective Picture System (IAPS; Lang, Bradley, & Cuthbert, 2008), which consists of 1,182 images and is designed for the experimental study of affective processing. It is based on the PAD model, involving pleasure/valence arousal, and dominance—a three-dimensional framework for measuring emotions (Mehrabian, 1996; Russell & Mehrabian, 1977). The validity of this theoretical model has accumulated a wealth of empirical evidence over time, and the number of citations for the database and instruction manual is now approaching 3,300, indicating a continued and robust research community surrounding it.

Using the IAPS database is particularly attractive due to the large variety of stimuli offered, as well as the chance to replicate and compare findings more easily between studies. Following the PAD model, each complete IAPS case is associated with normative (average) ratings for pleasure/valence (i.e., how positive or negative an image is), arousal (i.e., how alerting or calming an image is), and dominance (referring to the viewer’s perceived amount of control in relation to the stimulus displayed). To exploit the flexibility offered by such a large number of stimuli, several typical approaches for image selection have been used, with some of the most common being discussed below. However, it is important to note that most of these methods rely on assumptions about the underlying multidimensional structure of the database, and that violations of those assumptions can have profound consequences with respect to what inferences may be drawn from experiments using these stimuli. Specifically, if those assumptions are unsustainable, then some of the conclusions from the emotion literature may be questionable.

### Establishing group cutoff points

This method consists of selecting cutoff values, which usually divide one of the three continuous PAD distributions into different categories. For instance, Mikels, Fredrickson, et al. (2005) distinguished between positive and negative stimuli on the basis of which IAPS images had valence ratings above or below 5, respectively, given the rating scale used to measure PAD dimensions in the IAPS contains nine points. Similarly, Xing and Isaacowitz (2006) considered the images with valence scores between 1 and 4 to be negative, those between 4 and 6 to be neutral, and those over 6 to be positive, with images very close to these cutoff points being excluded (Xing, personal communication, June 6, 2015).

A variant of using group cutoff points is selecting extreme groups of images. This consists of retaining the first *n* most negative/positive images (or an upper and lower group of images), as well as a group with minimal distances from what is considered a “neutral” score. For instance, one of the four types of emotion induction used in Zhang, Hui, and Barrett’s (2014) study consisted of a combination of images and music, with some of the images being selected from the IAPS stimuli according to their rank (most positive, most negative, or most neutral).

Another extension of the cutoff point method was used by Lithari et al. (2010), who combined it with graphical presentation and selected images on the basis of how they were organized within a 2-D space. Four quadrants were formed through the crossing of the valence and arousal nine-point axes at a score of 5, and each quadrant was considered to represent a separate group of stimuli.

The cutoff point approach is best suited to research questions that focus on only one dimension of the PAD model. Although carefully chosen combinations of cutoff points may be adequate when a study focuses on only one or two dimensions, this strategy becomes unwieldy when researchers intend to systematically vary all three dimensions at the same time. Moreover, the use of cutoff points in this fashion tacitly assumes that the noncontrolled dimension(s) has (have) no effect on information processing or behavior that is relevant to the researchers’ interests—an assumption that is risky at the best of times. Finally, another implicit assumption, for which there appears to be no clear evidence, is that the groups formed using the cutoff points can approximate the internal structure of the IAPS data correctly.

### Discretization and crossing/controlling dimensions

This method refers to cutting the continuous PAD dimensions associated with the IAPS into *n* categories. Subsequently, within one such category, one may repeat the procedure on the basis of the remaining dimensions. For example, after cutting valence ratings into three categories, one may then attempt to find images of varying levels/categories of arousal within, for example, the most pleasant valence category. Alternatively, one may attempt to control one dimension within another—for example, finding one category with relatively constant arousal within the most pleasant valence category.

For instance, Tomaszczyk, Fernandes, and MacLeod (2008) chose IAPS stimuli on the basis of their valence ratings, but in addition attempted to cross different levels of arousal within the valence categories (see also Anderson, Siegel, & Barrett, 2011). Similarly, Aguilar de Arcos, Verdejo-García, Peralta-Ramírez, Sánchez-Barrera, and Pérez-García (2005) selected five categories of images for eliciting emotional experiences, including one neutral valence category with low arousal, and positive and negative valence categories, each with either a lower or a higher arousal level. Finally, Perri et al. (2014) divided the IAPS stimuli into positive, negative, and neutral categories based on their valence scores, with the first two of these categories presenting high levels of arousal. The neutral-valence pictures were selected to present low arousal.

If attempting to cross PAD dimensions in a factorial design in this manner, the assumption is made that the PAD dimensions are orthogonal (i.e., uncorrelated), which is not what the IAPS data suggest (Bradley & Lang, 2007). Similarly, attempting to control dimensions assumes that groups of images exist within the IAPS that vary in terms of one dimension, but not another. This is also generally not feasible, given that the correlated PAD dimensions tend to vary *together*. Finally, as is the case when using cutoff points, this method cannot easily accommodate the use of all three PAD dimensions simultaneously, usually resulting in dominance scores being ignored. Although it is correlated with the other two PAD dimensions, dominance represents a distinct entity within the model, and thus can itself account for some variation in affective ratings (Bradley & Lang, 1994). Therefore, if dominance scores are ignored, this variation would be excluded from the image selection process, which poses risks for its validity.

### Content selection

This type of stimulus selection based on content is usually combined with one of the previously discussed methods. For instance, Bernat, Patrick, Benning, and Tellegen (2006) selected erotic and adventure scenes as pleasant, and violent or threatening images as unpleasant stimuli. Neutral images were chosen to portray common objects or inactive people, and so on. In addition, this strategy was combined with dimension discretization/crossing, leading to groupings of pleasant and unpleasant images with low, medium, or high arousal levels (see also Tomaszczyk et al., 2008). In another study, Hamann, Ely, Hoffman, and Kilts (2002) selected IAPS images on the basis of their content: Pleasant pictures were chosen to depict erotic scenes, food, or agreeable animals and children. Negative images were selected thematically to include mutilated bodies, violence, and so forth. In parallel, high-interest images included exotic parades and surrealistic scenes, and low-interest images included plants or household scenes.

In addition, Eizenman et al. (2003) emphasized the thematic selection of IAPS images: Four categories were selected to include images considered neutral, dysphoric, threatening, or socially themed. However, the authors also relied on valence ratings to guide their selection procedure, so that neutral images were selected to have valence scores close to 5, threatening/dysphoric images ranged between valence scores of 2 and 4, and the social themes presented a range between 6 and 8 on the same scale. They also aimed to control variations in arousal levels by allowing maximum differences of two points across the images in each of the four categories. The content selection method does not place strong assumptions on the data on its own; however, it is usually used conjointly with the content selection, discretization and crossing/controlling dimension methods, which do.

### An alternative image selection method based on cluster analysis

The present work offers an alternative strategy for image selection based on clustering algorithms, which can be used with all three PAD dimensions simultaneously. To our knowledge, such algorithms have been used to categorize participant responses from individual studies (e.g., for classifying brain regions with differential response patterns to disgusting vs. neutral images—Deen, Pitskel, & Pelphrey, 2011; or for grouping participants in terms of their risk for alcohol abuse, on the basis of heart rate variability in response to IAPS emotional stimuli—Mun, von Eye, Bates, & Vaschillo, 2008), but not to group or select images on the basis of normative data.

In this article, we argue that clustering methods constitute a valuable means for creating experimental stimulus groups based on the IAPS normative data, by ensuring that group formation is optimized according to various measures (e.g., maximizing the distances between the different groups or the likelihood that cases belong to a certain group). This can boost the level of statistical power achieved in studies, since the larger the differences between levels of the treatment, the higher the chances of finding significantly meaningful effects (see Hallahan & Rosenthal, 1996, p. 495).

In addition to using more objective criteria for group formation, relative to entirely “manual” methods, clustering algorithms can also capture the particular structure of the IAPS data, and thus provide image classifications that are more empirically principled. This can allow experimenters to guard against confounds in the form of heterogeneous, systematically underpopulated, or “artificial” categories of stimuli, which cannot be adequately supported by the IAPS database. For instance, IAPS images are often divided into three groups based on valence. However, if this three-group structure is not an adequate fit for the IAPS normative data, images may be grouped inappropriately. Thus, if multiple types of negative material exist within the IAPS, creating only one category of negative images would risk blending these together, with unpredictable consequences for study results and the validity of any inferences based on them.

In addition, without consulting the structure of the IAPS data (which clustering methods are sensitive to), it might be tempting to resort to a factorial design combining three ordered levels of valence (low, neutral, and high) with as many levels of arousal. In this situation, it would be difficult to find enough images populating the intersection between low valence (i.e., negative images) and low arousal (i.e., relaxing images), due to the correlation between these two dimensions. Indeed, such a category could thus be deemed “artificial,” as it would ignore the essential correlations between PAD dimensions.

Consequently, clustering methods can provide information on both the quantity and quality of stimulus categories that can realistically be supported by the structure of the IAPS normative data. Although such algorithms can be flexibly adapted to extract a predetermined number of groups, usually they are allowed to follow an exploratory strategy constrained by the overall structure of the data set. That is, they will find the “best” number of stimulus clusters/groups, subject to some optimization constraints. This is a point of departure from the typical selection methods discussed above, in which a top-down process is often used to identify three image categories fitting the notions of “negative,” “neutral,” or “positive.” Finally, clustering algorithms can limit the amount of labor associated with stimulus selection, especially when research hypotheses involve more than one feature being taken into account at the same time (i.e., dominance, as well as valence and arousal). Indeed, by minimizing this difficulty, the method we propose below allows researchers to expand the scope and complexity of their hypotheses, and thus more easily test their theories.

Our hypothesis is that the IAPS data present a discernible, meaningful structure that can be capitalized upon by using cluster analysis to produce stimulus groups for experimental use. Here we tested several clustering approaches against one another, and propose a stepwise strategy for filtering and classifying IAPS images for subsequent experimental use. The family of clustering algorithms (or data-mining techniques) is extremely diverse and easily warrants entire books dedicated to them (for more detailed discussion, see Jain & Dubes, 1988; Kantardzic, 2011; and Kaufman & Rousseeuw, 2005). However, due to their widespread use and popularity, we focus on several approaches in particular. We will now briefly describe each of these approaches; readers interested in a more in-depth coverage may refer to the supplementary material.

The first approach is *k-means clustering*, which involves selecting *k* random seeds (i.e., random points in the space defined by the dimensions of the stimuli) and assigning the closest cases to them, leading to the formation of *k* groups. Afterward, the group mean (i.e., centroid) is computed, and cases are reassigned to groups on the basis of closeness to this value. This process will reiterate until the classification has settled into a stable solution (i.e., when the data points no longer change their memberships after the centroid computation). This is a hard partitioning method, meaning that all cases are included in their respective clusters with a probability of 1, and it does not provide a direct indication of the number of clusters existing in the data (Hartigan & Wong, 1979; MacQueen, 1967; Xu & Wunsch, 2009). Instead, various subsequent indices are used to suggest the number of clusters that would be appropriate for a given dataset. However, these do not take parsimony into account, and so may show little consistency or be prone to inflating the number of clusters. In order to establish clusters of images that could later be used as the levels of an “emotional content” independent variable, we tested *k*-means clustering because of its efficiency, simplicity, and wide use (Jain, 2010).

Another popular option is *hierarchical clustering*. This is an agglomerative method whereby individual cases begin by being designated as their own cluster (i.e., clusters of one data point each; Borcard, Gillet, & Legendre, 2011; Xu & Wunsch, 2009). Using one of multiple *linkage methods*, cases get merged progressively into ever-larger clusters, until all of the cases belong to just one, overarching cluster. Similarly to *k*-means, no indication is given about the suitable number of clusters in the data, so that with the aid of various statistical criteria (again not considering parsimony, and possibly conflicting in their recommendations), it is largely up to the researcher to decide where along this progression to stop and retain the corresponding number of clusters. Hierarchical clustering is also a hard clustering method, in which each case is assigned to one cluster exclusively, rather than being assigned a probability of membership.

A third option that is gaining in popularity is *model-based clustering*. This represents a form of hierarchical clustering that also involves an expectation-maximization (EM) procedure (for a primer on EM, see Do & Batzoglou, 2008). Unlike *k*-means, or hierarchical clustering per se, this is a soft clustering method, whereby cases are assigned to clusters with a certain probability (uncertainty) of membership. This can allow researchers to systematically control for the degree of typicality a stimulus exhibits in terms of the clustering dimensions used: A stimulus with higher uncertainty will be less representative of its cluster, and may introduce additional noise into experimental results. Also, in contrast with the two previous approaches, model-based clustering simultaneously provides both a clustering solution for the data and a straightforward method for determining the optimal number of clusters *k*. For this purpose, model-based clustering (implemented in the mclust R package: Fraley & Raftery, 2006) provides Bayesian Information Criterion (BIC) values and considers the optimal number of clusters for a given dataset to be whichever value of *k* maximizes^1^ this criterion. Therefore, one of the distinctive features of this method is that it takes parsimony into account in the attempt to reduce the unnecessary inclusion of components (clusters) into the model.

To summarize, in this article we focus on three types of clustering—namely *k-*means, hierarchical, and model-based clustering—each of which differs in the approach taken to assigning case membership (and whether that membership is probabilistic or absolute). Moreover, the first two approaches do not intrinsically provide a clear criterion for determining the final number of clusters, and so admit a variety of methods for deciding this (see below and in the supplementary material). We tested each of these methods on the IAPS data in order to: (a) gain more insight into the internal structure of the database; (b) identify any common patterns in clustering solutions across the different algorithms; (c) select the most suitable algorithm of the three and retain its clustering solution, and lastly; (d) extract a fixed number of representative IAPS images from the final clustering solution for use in further experiments.

Subsequently, we employed various validation techniques, to select one clustering method as the most appropriate for the IAPS dataset. After selecting one such clustering algorithm, we extracted the best exemplars from each resulting cluster, which we then propose as the final selection of stimuli that researchers may wish to use in subsequent work.

## Method

### Dataset characteristics

**Fig. 1:**
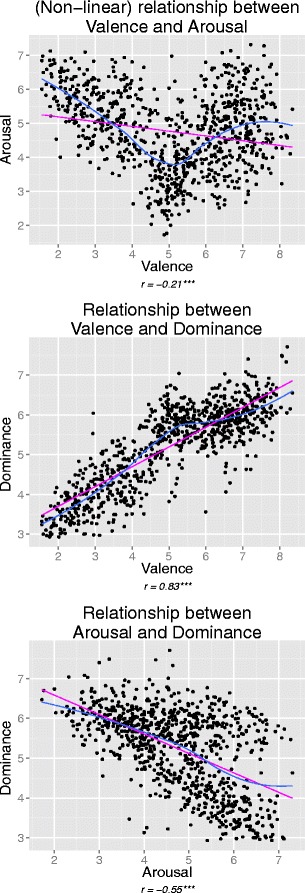
Correlations between the pleasure/valence arousal, and dominance dimensions, with deviations from linearity that give rise to the specific shapes of the relationships

In this study, we focused on the IAPS normative data gathered from both male and female participants, in which PAD ratings were collected using three (nonverbal) 9-point Likert scales (using the Self-Assessment Manikin, or SAM; Bradley & Lang, 1994; Lang, 1980) and a sample of approximately 100 US students, depending upon the image. In our analysis, we included all three PAD dimensions that are available within the IAPS data, to create stimulus groups that account for the maximum amount of variance in participant responses (Bradley & Lang, 1994). Despite the large correlations between dominance and the other two PAD dimensions (see Fig. 1), dominance did not perfectly overlap with them (e.g., if *r* > .9) either empirically or theoretically, further justifying its inclusion in subsequent analyses.

### Duplicates

We evaluated the univariate distributions available within the stimulus database, and identified 12 duplicate cases within the normative data (overall including *N* = 1,194 cases, but with *N* = 1,182 unique cases), each associated with different scores on the PAD model (see Table 1 for a listing). These images were likely normed twice, as part of different image sets (Lang et al., 2008). As a consequence, we replaced these duplicated pairs with a single entry containing the averaged valence arousal, and dominance across the duplicates.

**Table 1:** IAPS duplicates and their valence arousal, and dominance ratings (devised using the Stargazer R package; Hlavac, 2013)

Description	Image Code	Valence	Arousal	Dominance	Set
Spider	1230	4.09	4.85	4.58	1
Spider	1230	4.61	4.03	5.60	2
Horse	1590	7.18	4.74	5.54	2
Horse	1590	7.24	4.80	5.62	3
Rabbit	1610	7.82	3.08	6.77	1
Rabbit	1610	7.69	3.98	6.52	2
Coyote	1640	6.27	5.13	5.22	1
Coyote	1640	6.16	5.18	4.91	2
Cow	1670	6.81	3.05	6.53	1
Cow	1670	5.82	3.33	5.63	2
NeutFace	2210	4.38	3.56	5.03	1
NeutFace	2210	4.70	3.08	5.23	2
Mutilation	3000	1.45	7.26	2.99	1
Mutilation	3000	1.59	7.34	2.73	4
Mutilation	3010	1.71	7.16	2.88	2
Mutilation	3010	1.79	7.26	2.88	3
EroticFemale	4220	8.02	7.17	5.33	2
EroticFemale	4220	6.60	5.18	5.90	3
EroticMale	4520	7.04	5.48	5.48	2
EroticMale	4520	6.16	4.80	5.73	3
AimedGun	6200	2.71	6.21	3.35	1
AimedGun	6200	3.20	5.82	3.49	2
Exhaust	9090	3.56	3.97	4.51	2
Exhaust	9090	3.69	4.80	4.72	3

### Missing values

In terms of missing values, only the valence and arousal dimensions contained complete data. However, of the two dominance distributions (“Dom1” and “Dom2”)^2^ included in the database, depending on which SAM rating scale was used in the measurement (Lang et al., 2008), “Dom2” contained considerably more missing data than “Dom1.” Thus, we retained only the “Dom1” scale for further use,^3^ to benefit from its more complete data. We reduced the overall dataset accordingly, leading to a sample size of *N* = 942.

## Results

### Preliminary analyses

#### Outliers

Given the variety of emotional material included within the IAPS database, we employed a form of outlier identification as an objective means to filter out images exceeding the emotional intensity of stimuli expected in daily life, which could prove overly stressful for participants. Outliers might also distort the clustering solutions (e.g., for *k*-means and model-based approaches), thus constituting an additional reason to identify and remove them. Specifically, outliers used with the model-based clustering might lead to a different number of clusters and/or alter the cluster memberships, without necessarily nesting outliers into a cluster of their own (Fraley & Raftery, 2002; Hautamäki, Cherednichenko, Kärkkäinen, Kinnunen, & Fränti, 2005; Wu, 2012; Xu & Wunsch, 2009).

Using the R language (R Development Core Team, 2015),^4^ all three univariate distributions were found to be nonnormal according to the Shapiro–Wilk test, so any method of determining outliers that was based on averages would probably be inappropriate (since the averages would not adequately represent the distribution). Hence, we opted for a more robust indicator: the Median Absolute Deviation (MAD; Leys et al., 2013).^5^ Therefore, images that were more than 2.5 MADs away from the median, in either direction, were removed before further analyses could be conducted. No outliers could be identified using this method in the valence or arousal distributions, but interestingly, 32 images^6^ were flagged as outliers due to their dominance scores, and were thus removed. This was done to avoid distorting the clustering solutions subsequently, and also to filter out potentially harmful material, in an empirically principled, replicable manner.

#### Representativeness/precision of measures

Additionally, we implemented a measure to ensure the precision of the stimuli to be used: building 95 % confidence intervals (CIs) around the normative image ratings, to give an indication of how precisely the population means could be estimated, on the basis of the sample averages from the approximately 100 participants rating each image. We selected stimuli with CIs spanning no more than one point in total around the normative rating, which we considered to be sufficiently narrow, given that the three dimensions were measured on 9-point Likert scales. Using this criterion, 61 cases that were judged too imprecise were removed, since they could subsequently affect the inferences in our study; 46 images were removed due to the width of their CI on one dimension, 13 due to their CI width on two dimensions, and finally, two cases with CIs too wide on all three PAD dimensions simultaneously. After we had removed cases on the basis of both outlying values and CI widths, the sample size was reduced to *N =* 849.

### Clustering techniques

After employing the filtration methods described above, three clustering procedures—*k*-means, hierarchical, and model-based clustering—were used to produce a set of coherent clusters that could be used in later primary research. For the reasons explained previously, the clusters were built on the basis of the normative ratings for all three available measures associated with the IAPS: valence arousal, and dominance.

#### *K*-means clustering

**Fig. 2:**
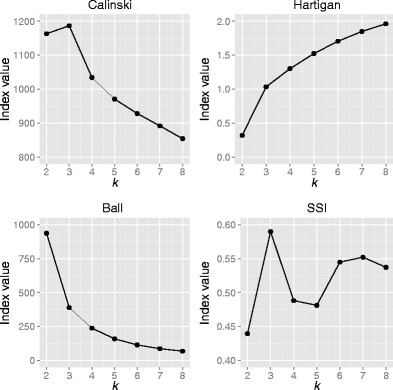
Various clustering indices indicate different “optimal” values for *k*. These graphs may change slightly with every run of the clustering algorithm, due to the random seeds that *k*-means uses. As such, 100,000 repetitions were run on the *k*-means clustering algorithm each time, with a range for *k* from 2 to 8, and with the values of the Caliński, Ball, Hartigan, and SSI criteria computed each time (with the Ball criterion having to be minimized, unlike the other three criteria, which must be maximized). The average values for these criteria were then computed across all of the repetitions and indicated (left to right, and top to bottom) that three, eight, eight, and three clusters should be extracted, respectively

When using this method, various indices were consulted to identify what the appropriate number of clusters (*k*) should be, including the Caliński–Harabasz Index (Caliński & Harabasz, 1974), the Ball Index (Ball & Hall, 1965), and the Hartigan Index (Hartigan, 1975), which are all based on within-/between-cluster sums-of-squares calculations (i.e., minimizing the former and/or maximizing the latter to ensure cluster compactness and/or the separation between clusters), as well as the Simple Structure Index (SSI; Dimitriadou, Dolničar, & Weingessel, 2002; Dolnicar, Grabler, & Mazanec, 1999), and others. The general trends shown by some of these indices are presented in Fig. 2, where the nature of the dataset is such that various clustering indices detect different characteristics of the data and do not converge on any simple answer as to the “correct” number of clusters that should be extracted. For further details on these and other indices, please see the supplementary material.

**Fig. 3:**
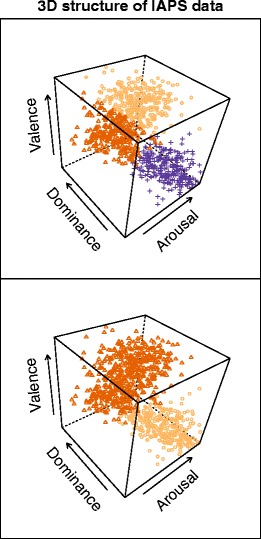
Data structure of the IAPS images. It is worth noting that large portions of the 3-D space remain unpopulated, signaling either that the IAPS does not cover those combinations between valence arousal, and dominance, or that photographic material in general would have difficulty with this

On the one hand, it may seem surprising that a subset of over 800 IAPS images may have several *k*-means clustering criteria peak for the number of only two^7^ or three clusters, considering the amount of variation in both the content and scores of the IAPS images. However, this could be accounted for theoretically by the emergence of a dichotomous “Positive and Negative Affect” structure (PA/NA, developed more in the Discussion), sometimes accompanied by the natural emergence of an additional neutral cluster. In Fig. 3, both clustering solutions are displayed using color coding for each cluster in the 3-D space, and are shown to cover extensive areas of the 3-D space.

**Fig. 4:**
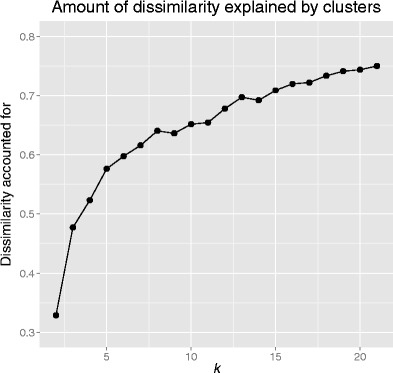
The amount of dissimilarity (as computed using the R package clue: Hornik, 2005) between cases is accounted for by ever-increasing values for *k*

On the other hand, higher values for *k* might be more suitable for the data, as is suggested in Fig. 4, which shows that as the number of clusters increases, so does the amount of explained dissimilarity between the cases (calculated as 1 – *unexplained dissimilarity* or 1 – *within-cluster dissimilarity*). Thus, as the number of clusters increases, within-cluster homogeneity also increases. However, *k*-means does not penalize for the increasing number of clusters (unlike model-based clustering), so that, conceivably, the total amount of dissimilarity would only be explained when the number of clusters equaled the number of cases. In other words, there is no single, definitive cutoff to determine which value of *k* best fits the data.

Since there may be arguments against using either a very small (e.g., *k =* 2 or even *k =* 3, with too many heterogeneous cases blended in the same group, as shown in Fig. 3) or a very large number of emotional categories (e.g., *k ≥* 8, leading to a very fragmented and unparsimonious structure, with relatively few cases per cluster), we now turn to the other clustering methods for additional solutions.

#### Hierarchical clustering

Jointly testing various linkage methods (i.e., strategies for progressively merging clusters, described in more detail in the supplementary material) and distance metrics allowed us to find the combination yielding the clustering solution with the highest degree of similarity to the original data (or matrix containing the distances between every pair of IAPS cases). We found that Average Linkage (i.e., merging clusters based on the average distance between their points) paired with correlation-based distances (i.e., assigning cases to clusters on the basis of correlations) produced the results most similar to the original distance matrix (cophenetic correlation, *r* = .91). Consequently, this combination was the most suitable for the IAPS data, and shows how essential PAD relationships are when determining how to group the IAPS images. The next best result was attained by Single Linkage (in which cluster-merging depends on the distance between the closest points belonging to different clusters), again combined with correlation distances (*r* = .87). Thus, after having reconfirmed the importance of the PAD correlations and identified the most suitable hierarchical agglomeration method for this dataset, we proceeded to determine the most appropriate number of clusters in the data.

In terms of connectivity, average silhouette widths, and Mantel optimality (briefly described within the supplementary material), a number of two clusters was suggested, whereas the Dunn Index indicated three. This corroborates the findings from some of the *k*-means indicators, and suggests the overall strength of the PA/NA structure within the IAPS, with or without an additional neutral cluster. However, as with *k*-means, some variability was to be found; for example, when using the elbow method for partitioning variance into clusters (using the GMD R package; Zhao & Sandelin, 2012), the optimal number of clusters (also based on average linkage) indicated was seven. Other clustering indices suggested nine clusters; however, still others provided more discrepant results, indicating numbers ranging from four to 15, or as many as 30 clusters. Overall, the most endorsed options were two (perhaps three), or nine clusters. For more information, please see the supplementary material.

#### Model-based clustering

Model-based clustering yielded a mixture model containing five clusters of varying Volumes, Equal (ellipsoidal) shapes, and Varying orientations (VEV). This model/configuration was optimal in terms of BIC values: BIC = –6,341.11, relative to the global minimum BIC value^8^ for other cluster numbers and configurations, BIC = –8,671.93 (for one spherical cluster, with either equal or variable volume, and the configurations abbreviated as EII and VII, respectively). The second best BIC value achieved was –6,343.72, for a VEV model with four components (clusters). Full details regarding the BIC values for all the models considered can be found in the supplementary material.

**Fig. 5:**
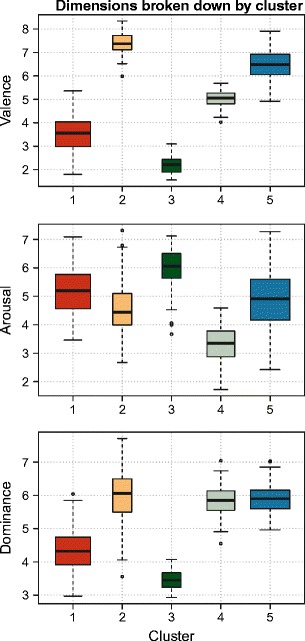
Cluster boxplots, for each dimension. The boxplots indicate, for each cluster (coded by colors), the spread of cases assigned to it, in terms of valence arousal, and dominance. The boxplot widths are proportional to the cluster sample sizes

The five-cluster solution proposed by the algorithm is described in Table 2, in terms of cluster centroids, sample sizes, mixing proportions (i.e., proportion of the mixture/overall sample that has been assigned to each cluster), and average uncertainties. By-cluster boxplots are also displayed in Fig. 5, comparing the relative spreads of the clusters’ valence arousal, and dominance univariate distributions. In addition, given the cluster centroids presented in Table 2, it is apparent that this clustering solution presents a symmetrical format: two negative clusters (one more so than the other), one neutral cluster, and two positive clusters (one more so than the other).

**Table 2:** IAPS cluster centroids, cluster sample sizes, mixing proportions (or the proportion of total cases assigned to each cluster), and average uncertainties, extracted using model-based clustering

Cluster	Valence	Arousal	Dominance	*N*	Mixing Prop.	Average Uncertainty
1	3.56	5.18	4.34	244	.29	.09
2	7.27	4.69	5.96	71	.08	.26
3	2.27	5.87	3.55	71	.08	.24
4	5.05	3.31	5.84	152	.18	.17
5	6.44	4.82	5.90	311	.37	.14

Finally, we assessed whether the assumption of multivariate normality held for these clusters, and found that, overall, the clusters presented ellipsoid shapes consistent with this assumption, with some further evidence also added by various multivariate normality tests. Please see the supplementary material for details on testing the assumptions required for model-based clustering.

### Validating the clustering solutions

After having employed three candidate methods—*k*-means, hierarchical, and model-based clustering—we proceeded to compare them on the basis of various validation techniques (full details are in the supplementary material), to select just one for further use. Given that variations were observed in terms of the “optimal” number of clusters suggested for *k*-means and hierarchical clustering by each clustering index, we deemed it appropriate to emphasize and pursue model-based clustering, which proved less affected by these issues, and also provided more information about the classification in the form of membership uncertainties. For a more meaningful comparison between the methods, parsimonious clustering solutions were formed using each of the three algorithms for a number of *k* = 5 clusters, as was suggested by model-based clustering.

#### Finding a stable structure within the data, across methods

Assuming that the IAPS data present a clear, discernible structure, all of the clustering algorithms should in principle be able to identify this structure despite their computational differences. To check this, we assessed the extent to which model-based clustering yields membership assignments that overlap with those from the other two competing methods.

The Variation of Information criterion (VI; Meilă, 2007) suggests that not much information is to be gained/lost when moving from one classification to another (i.e., there is considerable similarity between partitions of five clusters, regardless of the algorithm used to produce them), with the normalized VI between model-based and *k*-means clustering = .176 and the VI between model-based and hierarchical clustering = .217 (please see the supplementary material for details). This finding was corroborated by the relatively strong association found between partitions using Cramer’s *ϕ* (between the *k*-means and model-based classifications, *ϕ* = .704, and between the model-based and hierarchical classifications, *ϕ* = .516). Therefore, on the basis of the VI and Cramer’s *ϕ*, there is considerable similarity between the five-cluster solutions provided by the different algorithms. However, for further results, including those based on the Adjusted Rand Index (ARI; Hubert & Arabie, 1985), please refer to the supplementary material. Thus, on the whole these results constitute moderate evidence that a specific data structure can be identified in the IAPS, given the level of agreement between the clustering methods.

#### Evaluating the stability of the model-based clustering solution

We assessed the stability of the clustering solutions using various criteria, including split-half validation (i.e., dividing the IAPS data into two random halves and computing the level of association between the partitions created independently on these halves of the data) and jack-knife validation (i.e., removing 10 % of the IAPS data randomly across a few thousand repetitions and assessing changes in the structure of the clustering solutions). Overall, model-based clustering performed well, with a high degree of association present between how the random halves of the data were clustered, suggesting that the stimulus groups identified were well-supported. In terms of stability after the random removal of 10 % of the data points, model-based clustering also outperformed both *k*-means and hierarchical clustering, for which typically only one cluster was then identifiable in the data (i.e., no grouping of the data points could be achieved after the removal of data points using these methods). For more details on these and further analyses, please refer to the supplementary material.

### Selecting equal numbers of cases from each cluster

Given that the five clusters provided by model-based clustering differed in size, a procedure was required to sample equal numbers of cases from each cluster that would represent their respective cluster to the highest degree. Since levels of certainty are also provided for each image during the model-based clustering process, these could be used to create a hierarchy in terms of how likely it was for each image to belong to the cluster it was assigned to.

**Fig. 6:**
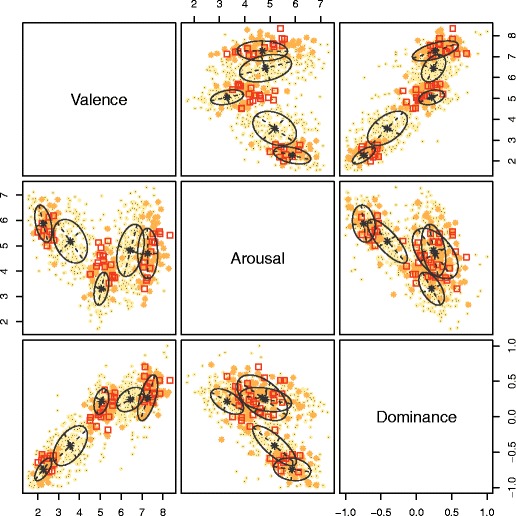
Bivariate scatterplots showing the default classifications of cases and the uncertainties provided by Mclust() in R. The uncertainties are coded using one of three symbols: ringed black dots for candidates with a high certainty of cluster membership; orange (light gray) asterisks for less clear cluster memberships; and red (dark gray) squares for cases to avoid using as stimuli, with very unclear memberships. Point size is an additional indicator for the level of classification uncertainty, with larger points indicating higher uncertainty

Consequently, a given number of images could be selected according to their rank in this hierarchy (i.e., the first *n* most likely cluster members). Figure 6 shows the default distinction made by Mclust(): Cases with uncertainties below the 75th percentile are considered acceptable, uncertainties between the 75th and 95th percentiles are risky candidates, and those over the 95th percentile should not be used, as they do not show clear membership to a given cluster. We made the same distinction in our final results, available online for download in the repository at www.github.com/CaterinaC/IAPSClustering2016, where we indicate which IAPS images were assigned to which cluster, as well as the level of uncertainty associated with this classification—particularly, which uncertainties were above or below the 75th percentile (i.e., whether or not they should be sampled for research). These results are suitable for researchers to use in most research contexts.

**Fig. 7:**
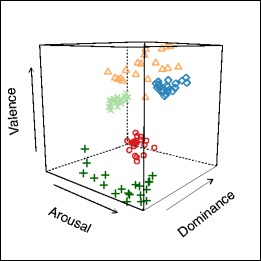
Selection of the 20 most likely IAPS cases per cluster, for the *k* = 5 clustering solution. The color coding was chosen to be consistent with Fig. 8 below

**Fig. 8:**
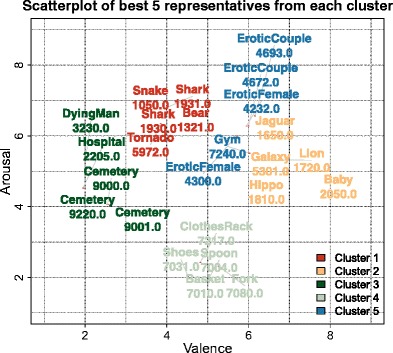
Selection of the five most likely IAPS cases per cluster, for the *k* = 5 clustering solution, along with IAPS image codes. The color coding was chosen to be consistent with Fig. 7 above

In our example, only the first 20 cases in the hierarchy of uncertainties were retained for closer inspection. These can be judged as the best representatives for each given cluster, and are portrayed in Fig. 7, with the first five of each cluster also displayed in Fig. 8, where they are shown to be meaningfully related to one another.

### A comparison between our method and ad-hoc approaches to selecting IAPS stimuli

Studies relying on more typical, ad-hoc methods for sampling IAPS stimuli may face several risks. On the basis of a Google Scholar search for “IAPS images,” we selected a small number of studies randomly from several pages of results. However, we only retained articles that also specified the IAPS image codes used, rather than simply the average PAD values for the images selected. We then assessed how the categories used in these studies matched our own.

First, as is shown in Table 3, the images intended to represent different affective categories in these studies sometimes share the same clusters that our model-based clustering uncovered. For instance, in the Glenn, Blumenthal, Klonsky, and Hajcak (2011) study, four images considered neutral and ten images considered pleasant all belong to one of our *positive* clusters (i.e., Cluster 5; see also Table 2 for cluster descriptions).

**Table 3:** Stimulus groups used in various studies, redistributed according to our method

Study	Stimulus Categories Used	Total No. Stimuli Used per Category	Image Codes Unaccounted For	Stimulus Redistribution According to Our Method, According To:							
				Missing Dominance Score Excluded	Outliers Excluded	Wide CI Excluded	C1 (−)	C2 (++)	C3 (− −)	C4 (±)	C5 (+)
Glenn et al., 2011	Pleasant	18	–	–	–	3	–	5	–	–	10
	Neutral	18	–	1	–	–	–	–	–	13	4
	Unpleasant	18	–	–	6	3	4	–	5	–	–
Mikels, Larkin, et al., 2005	Pleasant	80	17	–	–	10	–	22	–	–	31
	Neutral	80	8	–	–	–	7	5	–	41	19
	Unpleasant	80	–	–	3	2	71	–	4	–	–
Most et al., 2005	Neutral	55	–	–	–	–	8	–	–	27	20
	Negative	39	–	–	11	6	5	–	17	–	–
Stins & Beek, 2007	Neutral faces	14	–	1	–	–	2	–	–	10	1
	Neutral household items	15	–	1	–	–	–	–	–	14	–
	Erotic	17	–	1	–	7	–	–	–	–	9
	Family scenes	12	–	1	–	2	–	8	–	–	1
	Mutilation	11	–	–	6	–	–	–	5	–	–
	Fear	18	–	1	5	1	9	–	2	–	–
Koenigsberg et al., 2010	Negative	47	–	6	7	4	13	–	17	–	–
	Neutral	49	–	10	–	–	4	–	–	25	10

The clusters from one through five are represented by C1, C2, C3, C4, and C5, and refer to those described in Table 2. For the same columns, between parentheses we have included concise information about the valence of our clusters, ranging from very positive (++) to very negative (− −). This has been done merely to aid interpretation of this table; however, as we previously stated, all three of the PAD dimensions were important in determining how the IAPS stimuli were assigned to these clusters, not only valence.

Second, the negative or positive stimulus groups used in studies tend to pool together stimuli that our method has distinguished as reflecting two types of positive or of negative material. For example, the Koenigsberg et al. (2010) study used a group of stimuli wholly considered to be negative; however, our method divided these between two separate clusters—one that is mildly negative and moderately arousing, and one that is more negative and more arousing, and with lower dominance than the former cluster.

In some cases, a single stimulus category (i.e., neutral, on the basis of the research reviewed in Table 3) may spread across three or four of our clusters. For instance, in the study by Most et al. (2005), the neutral category in fact included eight mildly negative images, 27 neutral images, and 20 mildly positive images, according to our method. Another example is the study by Mikels, Larkin, Reuter-Lorenz, and Carstensen (2005), in which a category of neutral images intended to differ only in brightness actually belonged to four different emotional clusters within our own classification.

In addition, from Table 3, it is also apparent that without filtering images on the basis of 95 % CIs, less reliable image stimuli can be included in studies. For instance, in the case of the Stins and Beek (2007) study, seven less reliable (in terms of confidence interval widths) images were included in the group of erotic stimuli. Similarly, images have also been selected without taking into consideration dominance—including some images we excluded precisely because their norms for dominance were missing. Finally, IAPS data outliers have also been included in studies, which could pose some ethical risks, due to their emotional intensity, and warrant closer inspection.

## Discussion

A variety of research areas rely on stimulus databases for experimental use. The IAPS is one such widely used database, having currently amassed approximately 3300 citations in Google Scholar (April, 2016). Yet, despite its extensive use, a standard stimulus selection strategy from the IAPS has yet to be devised—one that can easily take into account all three PAD dimensions simultaneously, and provide a stimulus grouping that is both empirically principled and optimal in terms of various statistical measures.

In this article, we proposed such a method based on the following sequence of steps: filtering out stimuli that constitute outliers or duplicates, and those with CIs wider than a preset criterion; creating stimulus categories using different clustering algorithms; and finally, validating these categories against several measures. Within the procedure we propose, we placed special emphasis on model-based clustering, an inferential method that provides not only a classification of the stimuli, but also an uncertainty estimate for each stimulus assigned to a cluster. Examining these uncertainty estimates allows researchers to control for how well stimuli reflect their underlying category and to select only those stimuli that reflect their cluster in the most meaningful way.

### Filtering out stimuli prior to clustering

As a first step toward creating a selection of stimuli for experimental use, the MAD has proved to be a useful tool for identifying stimuli that may be ethically questionable, due to their violent or threatening nature. In addition, Grühn and Scheibe (2008) found that IAPS ratings for negative images tend to get more extreme with age. Thus, as a precautionary measure, filtering out outliers using the MAD might have to be considered more carefully depending on what sample/population the stimuli are aimed at, as the same IAPS image might be more distressing for one category of participants than another.

Using the MAD, we were able to exclude 32 images due to their particularly low dominance scores (i.e., in the case of highly violent images, with an average valence level of 1.98—e.g., image 3001, a headless body; 3131, mutilation; 3170, a baby with a tumor; etc.). Interestingly, these same cases were not flagged as outliers given their scores on the other dimensions. This provides further evidence that dominance scores reflect a different process of emotional evaluation and should be considered more frequently when selecting IAPS images. Relatedly, dominance is believed to be more easily distinguishable from the other two dimensions in social situations (rather than photographic material; Bradley & Lang, 2007, p. 32), further supporting its general inclusion in stimulus selection procedures, as an additional contributor to emotional experiences.

The large standard deviations associated with the ratings for most stimuli from the IAPS have usually resulted in wide 95 % CIs (spanning more than one point on the nine-point Likert scale used for ratings). However, within our overall approach based on CIs, other (more or less conservative) criteria may also be applied regarding the width of these CIs, depending on researchers’ specific aims. This type of verification has proven to be highly useful either for deciding which stimuli to retain for the subsequent clustering procedure, and for a better appreciation of the amount of variability in the individual IAPS ratings leading to the normed means. Although we are unable to give an exact reason why some of the stimulus norms were insufficiently precise, on the basis of our criterion, these results clearly suggest a verification as simple as this should become a more standard practice when selecting stimuli from stimulus databases.

We would stress that it is possible for any emotional stimuli database to present these same concerns. This is because emotional stimuli are conceivably very subjective, thus leading to the large standard deviations observed, and implicitly, the lower degree of certainty as to how they may be perceived by individual participants (e.g., image “EroticFemale” 4210 registered the highest standard deviation of all IAPS images, suggesting that reactions to it varied considerably). On the other hand, it is also possible these characteristics might be specifically related to the features of IAPS, but not of other emotional stimuli collections; thus, image quality and historic context, ecological validity, and so forth, may also be involved. Future work will be necessary to address this research question.

### Clustering the stimuli

When using *k*-means and hierarchical clustering to classify IAPS images, the repartition of cases between clusters represents a separate step from choosing the “appropriate” number of clusters existing within the data. Our analysis showed that it is difficult to discern a clear cluster structure within the IAPS data. For example, in the case of *k*-means, the optimal value for *k* oscillated between two, three, or eight, depending on the clustering index used, and on the total number of clusters tested. Similarly, for hierarchical clustering, a number of two, three, seven, or nine clusters was indicated as suitable for the IAPS data, also depending on the index and number of clusters. It may seem surprising that a number of clusters as low as two, or even three, could be suggested by both *k*-means and hierarchical clustering, for a sample size as large as *N =* 849 images, varying considerably in terms of valence arousal, and dominance scores. However, the emergence of these solutions is understandable, for theoretical reasons and/or due to the shape of the IAPS data.

First, the *k =* 2 solution carries theoretical significance by corroborating principles used in the construction of the Positive and Negative Affect Schedule (PANAS; Watson et al., 1988), since the two emerging clusters can be interpreted as matching the Positive and Negative Affect components of the scale, which measure the corresponding affective moods with adequate reliability and validity. This similarity directly indicates that clustering methods can provide meaningful results, which can be validated against current practices and/or theory.

Second, the nonlinear (“U” shaped) relationship between valence and arousal can easily be split into three sectors, a characteristic that carries over into 3-D space, when dominance is added. Thus, one cluster is negative with higher arousal, another is neutral with lower arousal, and the third is positive, again with higher arousal. Although this three-cluster solution may appear similar to those from typical image selection practices (cutoff points and/or factorial designs, centered on selecting three valence groups: negative, neutral, and positive), it differs from these approaches in that it accommodates all three PAD dimensions simultaneously with ease, and also takes the structure of the data into account, without imposing unsustainable assumptions (i.e., independence of the PAD dimensions). In fact, even if hierarchical clustering did not provide the final classification of the IAPS data, it did reveal most clearly the importance of the PAD relationships, since using correlation-based distances always yielded the highest correlations with the original data for this clustering method. This suggests that the PAD correlations should always be taken into account when selecting stimuli from the IAPS, whereas using factorial designs without concern for them may simply lead to inappropriate groupings of stimuli, and subsequent experimental results that are difficult to interpret.

However, both of these solutions (*k* = 2 and *k* = 3) focus on the creation of just a few, large clusters, which would thus cover considerable portions of the 3-D affective space within the PAD model. As such, one large negative cluster would, for instance, include images with both moderate and higher arousal, or both moderate and lower dominance—leading to a lower degree of experimental control.

On the other hand, from a practical standpoint, the larger numbers of clusters (seven, eight, or nine) indicated by *k*-means and hierarchical clustering may be as intractable as the lower numbers, but for different reasons. Rather than blending together too many heterogeneous cases, when using a larger number of small clusters—the more clusters are extracted, the closer their centroids necessarily become and thus their “best representatives” are also drawn nearer. This can result in a potential reduction in statistical power. Also, more clusters (or treatment levels) would generally signify longer testing times and study expenses, which is not always feasible. Finally, smaller cluster sizes would be less useful for experiments requiring larger numbers of stimuli of the same type (i.e., from the same cluster).

In contrast to the previous two methods, model-based clustering uses a soft clustering approach, which provides an estimate for the degree of cluster membership (uncertainty) associated with each image. This allows for finer-grained control over stimuli used in experiments, which in turn can help make research inferences stronger. This method also provides additional flexibility in terms of adaptively distinguishing a variety of cluster configurations, thus being capable of a closer fit to the original data. In contrast, *k*-means would, for instance, favor spherical clusters in particular (Jain, 2010). Finally, unlike for *k*-means or hierarchical clustering, the optimal number of clusters in model-based clustering is assessed using the BIC, which penalizes for large numbers of clusters, and simplifies the process of choosing which number of clusters to extract from the data.

In our case, a number of five clusters was suggested, which also represents a good compromise from a practical standpoint. In addition, the clusters were determined to be of Varying volumes, Equal shapes (i.e., ellipsoidal, rather than spherical), and Varying orientations within the 3-D space. The cluster centroids also suggest that for participants, “neutral” images present medium levels only on the valence scale, rather than in the whole PAD model, as might have been assumed. Thus, neutral IAPS images tend to be somewhat lower in arousal and higher in dominance: For instance, a picture of a mug (IAPS code 7035) intuitively seems “neutral,” but this translates into medium values only on the valence dimension (norm = 4.98), whereas the lower arousal (norm = 2.66) suggests a more calming influence, and the higher dominance (norm = 6.39) suggests very unchallenging content.

Equally, we have shown that two forms of negative and positive material exist, rather than one of each, which is the typical grouping used in research. For instance, we found that very negative content (e.g., “Mutilation”, IAPS code 3030) presents very low valence (as expected) but, uniquely, higher arousal and lower dominance. Thus, collectively, these three components (and not just valence) seem to form what is usually perceived as “very negative” content. A second, milder, type of negative content was identified, as well, which still presents valence values below the scale midpoint, but less extreme arousal and dominance values (e.g., “Cigarettes”, IAPS code 9832). Similarly, positive content can also be divided into two subtypes using our method: positive, more arousing content (e.g., “Erotic Couple”, IAPS code 4693) and very positive, more serene/less arousing content (e.g., “Nature”, IAPS code 5220)—with both of these categories being fairly similar in their mean-level dominance.

This five-cluster option generally benefits from empirical support based on the methods we employed to verify this. We first noted a moderate overlap between how the images were classified into five groups by *k*-means, hierarchical, and/or model-based clustering, depending on the measure used to assess the overlap. Although no structure is unanimously accepted within the IAPS data, measures such as the Variation of Information (VI) or Cramer’s *ϕ* both suggested that *k* = 5 is relatively well-supported, even if each clustering method can shed its own perspective on the data (i.e., the amount of overlap was not maximal, which we discuss in more detail in the supplementary material).

Subsequently, to ensure that model-based clustering is indeed the most suitable algorithm for use with the IAPS data, we removed 10 % of cases randomly across a few thousand repetitions (using jack-knife validation), each time assessing how the optimal number of clusters changed. Ideally, if a robust clustering solution was found using a certain clustering algorithm, the removal of 10 % of the values should make little difference. In the case of *k*-means and hierarchical clustering, this frequently resulted in only one all-encompassing cluster being identified in the data, which was deemed inappropriate. In contrast, model-based clustering showed more stability, and most often suggested *k* = 3 (followed by *k* = 4) as the optimal solution in this case. However, cross-tabulations showed that these model-based solutions were very closely correlated to the *k* = 5 solution achieved on the full dataset, and did not present any deeply concerning changes such as the cluster structure collapsing entirely (i.e., when we found just one cluster using the other two methods). Therefore, the differences seen in the values of *k* most likely reflect the fact that one or two clusters from the *k* = 5 solution were collapsed due to the induced data attrition (–10 %), but that similarities between the solutions nevertheless remained robust.

Finally, when predicting the clustering structure of a random 50 % of values based on that of the other 50 % (using split-half validation), and comparing this prediction to the observed model-based classification of the target half, the two matched very closely. On the basis of all these indicators, we concluded that the five-cluster mixture model is well-supported by the IAPS data.

### Method summary and recommendations for use

As an outline for our method, we recommend first inspecting the IAPS images and filtering out duplicates, outliers, and images with CIs larger than a preset criterion (we opted for one point in total, on the Likert scales used for the IAPS norms, but researchers may be more conservative if they have specific reasons for this). Subsequently, on the basis of the findings detailed above and in the supplementary material, we recommend resorting to a model-based clustering algorithm, which will nest the remaining images into five clusters, while also taking into account arousal and dominance in the creation of these clusters, even if researchers may only be explicitly interested in, for instance, valence.

Regarding any more practical issues that may arise, we recommend maintaining this well-supported, five-cluster structure even if researchers may be interested in comparing fewer categories. For instance, assuming that a study is aiming to compare the effects of positive versus negative valence on an outcome variable, just two of the five clusters may be used, which are farthest apart on this dimension, rather than altering the clustering solution to provide just two clusters in total.

Given that model-based clustering is a soft clustering method, cases were also assigned a level of certainty for belonging to their cluster. Unequal cluster sizes (with some of them being perhaps too large to be used in an experiment in their entirety) led to cases being sorted in descending order of their certainty of membership. This enabled us to select a constant number of images per cluster for subsequent use in an experiment—those at the top of the hierarchy formed (i.e., with the highest certainty of membership, or equivalently, with the lowest uncertainty). Besides providing the ability to flexibly tailor this constant to the requirements of individual studies, these stimuli can also act as the best representatives of their respective clusters.

For illustrative purposes, five to 20 cases per cluster were sampled in the order of their certainty of belonging to their given cluster. This resulted in groups that are intuitively meaningful, with one very negative cluster including death-related scenes (e.g., hospital, cemetery, dying man); a second negative cluster including dangerous agents, which was higher in dominance than the former one (e.g., snake, bear, shark); one neutral cluster that was low in arousal and higher in dominance (e.g., spoon, shoes, basket); one positive cluster including arousing scenes (e.g., erotic scenes, gym); and finally, another very positive cluster including less arousing “natural” scenes (e.g., hippo, jaguar, galaxy).

Depending on the number of stimuli required per cluster for individual studies, researchers may also wish to know how many stimuli can safely be sampled from the clusters, in their order of membership certainty. One solution could be to use the criteria from the default Mclust() (Fraley & Raftery, 2006) graphical output in R, which considers images with uncertainties below the 75th percentile to be appropriately clustered. Of course, more conservative cutoffs could be selected, should the amount of data support it, the number of stimuli required be relatively small, or the study imply high stakes (e.g., in clinical research).

If, on the other hand, researchers require larger numbers of images per cluster than, for instance, those having uncertainties below the 75th percentile, or even more than the size of the smallest clusters extracted (e.g., *N* = 71, in our case), several solutions exist. First, one can relax the reliance on uncertainties when excluding images, but nevertheless retain the uncertainties for use as statistical weights in models, after experimental data have been collected. This would ensure that better cluster representatives would count more when determining the research results, making images with higher uncertainties still usable. A second alternative could be to resort to sampling additional photographic stimuli from other databases. To the extent that PAD ratings/norms exist or can be obtained for such images, it would be trivial to determine their cluster memberships with regard to the present results.

Finally, it is also possible for researchers to modify our method to suit their aims—for instance, in terms of the criteria used for the CI widths, or the level of uncertainty used to determine clear cluster memberships—as long as there is good justification for doing so and deviating from the standard approach (e.g., in clinical research with high stakes).

### A comparison between our method and ad-hoc approaches to selecting IAPS stimuli

On the basis of our brief comparison, we discovered that a common practice is to group together stimuli that, according to our method, actually represent different types of negative or positive images (e.g., when a single group of positive material is used, instead of one positive cluster of “serene scenes,” with lower arousal and somewhat higher dominance, plus one cluster of “exciting scenes,” with higher arousal and somewhat lower dominance). Thus, a single, generic grouping of “positive” (or “negative”) images may obscure any specific effects due to just one *type of* positive (or negative) material—particularly if the effects actually differ between the several types of positive (or negative) images.

This would be in addition to the relatively frequent inclusion of outliers in the literature, and importantly, of less reliable images (with 95 % CIs wider than one point). Of these, outliers could be ethically risky, and should be avoided especially when relying on cluster analysis for stimulus selection (otherwise, they may distort the clustering solutions), whereas images with wide CIs can introduce additional error variance into research results.

Another interesting finding that emerged from our comparison is that effects can become diluted if neutral categories are not truly neutral, and extend into the space of clusters that we have found to actually be mildly positive or negative. This could result in diminished power to detect differences between the “neutral” and positive or negative stimulus categories.

Finally, we would underline that we do not wish to highlight these differences as criticisms of previous research using the IAPS. Rather, it is our intention to improve on these very widespread methods for selecting stimuli, by promoting our novel method that relies on model-based cluster analysis. Indeed, we believe previous image selection techniques may still be useful in limited contexts; however, it would be very difficult to predict when or to what extent they might influence results (by obscuring effects or “diluting” them, etc.). In addition, they may often vary considerably from study to study (in terms of both selection criteria and resulting selections), making comparisons between studies more difficult. As such, we argue that relying on a statistical, easily reproducible^9^ and automatic procedure, which also quantifies the extent to which images belong to a given cluster, is much to be preferred.

### Further research and limitations

Despite being arguably more objective than “manual” selection methods, cluster analysis is not an “exact science.” As has been shown previously, the large variety of algorithms available can lead to substantial variations in clustering solutions. It is sometimes partly up to the researcher to decide which clustering solution is appropriate for their data. This is particularly the case with *k*-means and hierarchical clustering, because the clustering process is initialized using random seeds and/or various clustering indices that may suggest conflicting numbers of clusters. In contrast, with model-based clustering such difficulties can largely be avoided, because the results are identical on different runs of the algorithm (unlike *k*-means), and the only relevant criterion for choosing the number of clusters is the BIC.

Thus, any flexibility attributed to clustering methods (model-based clustering, in particular) may be seen as an asset, rather than a risk for objectivity, as long as the choices made by researchers (i.e., level of uncertainty, the width of CIs, etc.) are transparent and justified by convincing arguments. The present work aims only to provide a guide for a method that is more appropriate than manual selection strategies—particularly if multiple dimensions are used simultaneously for selecting stimuli.

In addition, although the cases sampled from each cluster acquit themselves of being good cluster representatives, the overall selection of treatment levels (or clusters) is ultimately constrained by the type of data in the IAPS—or whichever stimulus database would be used in research. As such, the final selection of stimuli cannot include categories of stimuli that are not part of the database to begin with. In the case of IAPS data, this may be either because such stimuli would be difficult to find, due to the PAD correlations (e.g., very negative images with low arousal are unlikely), or because the IAPS domain of images does not include emotional material that extends as far as possible within the 3-D PAD space (e.g., images with moderate valence and moderate, rather than low, arousal are not very common).

These concerns could be addressed in the future either by the inclusion of new images or by a renorming process for the IAPS database (potentially via Amazon Mechanical Turk), using larger samples to rate each image. This can also present the added benefit of the average values being more stable (i.e., smaller standard deviations), and therefore fewer images being filtered out of the clustering procedure, thus creating more comprehensive clusters. However, until then, when interpreting results based on the current IAPS norms, the empty areas in the PAD space will require careful consideration, since otherwise research conclusions may be biased.

In terms of future research, an interesting avenue would be to compare empirical results when using a manual image selection method, relative to our cluster-analysis-based classification. Also, there is room yet for further standardization of the IAPS images—for example, in terms of their spatial frequency content (i.e., their level of detail or “coarseness”), which may interact with their affective processing (Delplanque, N’diaye, Scherer, & Grandjean, 2007). Cluster analysis could take such dimensions (as well as participant age, etc.) into account when creating experimental treatment levels, provided they have been converted to standard scores beforehand. Furthermore, depending on whether the raw data used to produce the IAPS normative ratings will be made available, the source of the large standard deviations could be explored further, to indicate improved selection strategies.

Finally, for any research requiring “emotionally ambiguous” stimuli, which do not clearly fit into any particular cluster, uncertainty estimates for the classification of images may provide a more empirically principled means to identify these along multiple dimensions. This would represent a higher level of rigor, the application of which could be explored in future research.

### Conclusions

In this article, we have presented a method for selecting experimental stimuli, which we have illustrated using the IAPS database. Using model-based clustering and valence arousal, and dominance scores, we classified the IAPS images into five categories—with each image presenting a certain level of certainty of belonging to its respective cluster. Our method is flexible, efficient, and reproducible, and it provides meaningful clusters in a symmetrical format, in terms of their valence ratings: two negative clusters (one more so than the other); one neutral cluster; and two positive clusters (one more so than the other). However, this method could easily be extended to other stimulus databases, in which the same principles may be applied: careful data inspection, including the removal of any duplicated cases in the stimulus database; the exclusion of missing values and outliers (in a judicious manner); selecting the most precise cases; selecting an appropriate clustering algorithm and clustering solution; and finally, extracting a constant number of stimulus exemplars from each cluster.

## Electronic supplementary material

Below is the link to the electronic supplementary material.

ESM 1 (DOCX 824 kb)

## Footnotes

1 The formula employed by Fraley and Raftery (2006) uses the negative of deviance, so that BIC here needs to be maximized rather than minimized, which is more common.

2 “Dom1” refers to the “classic” SAM dominance scale, whereas “Dom2” refers to a version of the dominance scale on which the SAM icon with the highest control presented a more assertive and dominant facial expression/posture than in the classic version.

3 The correlation between “Dom1” and “Dom2” was remarkably high (*r* = .98), for the *N* = 60 cases measured on both versions of the dominance scale. Thus, we were able to safely use only “Dom1” in our analyses.

4 The R code for our analysis is available at www.github.com/CaterinaC/IAPSClustering2016.

5 According to this method, acceptable values should lie between the median ± (*x* * MAD), where we opted for *x* = 2.5.

6 IAPS codes: 3000, 3001, 3010, 3015, 3053, 3059, 3063, 3064, 3080, 3102, 3131, 3170, 3266, 3500, 3530, 6230, 6231, 6250, 6250.1, 6260, 6263, 6300, 6350, 6510, 6520, 9075, 9252, 9410, 9413, 9600, 9908, and 9940

7 Please refer to the supplementary material for more details on the measures of Connectivity and Average Silhouette Width that suggested this value.

8 Mclust() seeks to *maximize* BIC values, given that it uses the negative of deviance.

9 As long as any researchers using model-based clustering are transparent about all of the settings/data-cleaning methods used with the procedure.
