# Supplementary material for: A cluster-based approach to selecting representative stimuli from the International Affective Picture System (IAPS) database
Source: Behav Res Methods. 2016 Jun 10;49(3):896–912. doi: 10.3758/s13428-016-0750-0 (PMC5429379; doi:10.3758/s13428-016-0750-0)
Supplement: Supplementary file 1 — (DOCX 824 kb) [file 13428_2016_750_MOESM1_ESM.docx]

**A Cluster-Based Approach to Selecting Representative Stimuli from the International Affective Picture System (IAPS) Database**

**- Supplementary material -**

Alexandra C. Constantinescu, Maria Wolters, Adam Moore and Sarah E. MacPherson

# Preliminary analyses

**Representativeness / precision of measures**. A further screen – the coefficient of variation (*cv*)^[[1]](#footnote-1)^ – was used alongside the 95% confidence intervals to assess how representative the normative ratings are for the sample distributions they were computed from. Normally, a measure of variability tailored to ordinal data would have been most appropriate here, such as the Index of Ordinal Variation, or the Coefficient of Ordinal Variation (see Berry & Mielke, 1992; Blair & Lucy, 2000; Kvalseth, 1995). However, its application is not possible without the raw IAPS data forming the norms - which are not made available. Thus, the *cv* was used instead, as it relies only on the IAPS norms/means and standard deviations provided.

We aimed to exclude stimuli with unrepresentative means (i.e., with *cv* > 30, Brown, 1998), but this measure proved to be far too conservative for the IAPS dataset, given that only one case complied with this criterion simultaneously for all three PAD scales, out of the *N*=849. Consequently, only the criteria based on outlying values and confidence intervals were maintained, as otherwise using the coefficient of variation would have resulted in the exclusion of nearly all stimuli, except one.

# Results

## Clustering techniques

***K*-means clustering.** Euclidean distances were chosen to create the *k* partitions^[[2]](#footnote-2)^, and various clustering indices were calculated to assess the suitability of extracting between two and eight clusters. These indices were offered by the clustIndex() function from the cclust R package (Dimitriadou, 2014), and included: the *Calinski-Harabasz Index* (Caliński & Harabasz, 1974), the *Ball Index* (Ball & Hall, 1965), the *Hartigan Index* (Hartigan, 1975) and the *Simple Structure Index* (SSI, Dimitriadou et al., 2002; Dolnicar et al., 1999). The first three of these are based on within-/between-cluster sums of squares calculations (i.e., minimizing the former and/or maximizing the latter to ensure cluster compactness and/or separation between clusters), whereas the fourth is a composite measure taking into account: the maximum difference between clusters, the size of the most different clusters, and the difference between cluster centroids and the grand mean of each dimension. For details, please refer to Table S1.

In addition, we consulted the clValid R package (Brock et al., 2008) in order to assess the optimal value of *k* in terms of internal validation measures. Thus, using Euclidean distances, we computed: the *Dunn Index* (Dunn, 1973), i.e., the ratio between the smallest distance between cases assigned to different clusters, and the maximum distance between two cases assigned to the same cluster; the *measure of Connectivity* (Handl et al., 2005), i.e., the extent to which neighboring data points share cluster membership; and the *Average Silhouette Width* (ASW, Rousseeuw, 1987), i.e., the extent to which data points are closer to other points in their own cluster, rather than the nearest points assigned to a different cluster. For details on these indices, please refer to Table S1. Overall, the Connectivity, Average Silhouette Width, Calinski-Harabasz Index and Simple Structure Index pointed to a lower number of clusters: two (in the case of the former two indices) or three (the latter two indices), whereas the Hartigan, Ball and the Dunn Indices, all pointed to eight clusters, the maximum number tested.

Finally, we also used the NbClust R package (Charrad et al., 2014) which computes thirty such indices and indicates which value of *k* is considered to be optimal, of those tested. Thus, when testing each option between *k*=2 and *k*=15, twenty of the clustering indices recommended to extract either two or three clusters, with the remaining indices suggesting that four, ten, fourteen or even fifteen clusters (the maximum tested) be extracted. In order to observe the behaviour of the algorithms, we also extended the number of clusters tested to thirty, rather than fifteen, and re-assessed the clustering indices. In this case, the majority of indices (i.e, seventeen out of thirty) again suggested to extract either two or three clusters from the data, with the remaining indices recommending four, ten, fifteen, seventeen or even thirty (again, the maximum number tested).

Thus, it is apparent that two diverging trends exist within the IAPS data: on the one hand, a tendency to group a large number of images in just two or three clusters, and on the other, a tendency to achieve a finer-grained grouping - although in this case, the number of clusters can vary considerably by the index used, with one or two clustering indices endorsing each of: four, ten, fourteen, fifteen, seventeen or even thirty clusters in the data, depending on the maximum number allowed in the search.

**Hierarchical clustering.** This is also a hard clustering method, where each case is assigned to exclusively one cluster, rather than being assigned a probability of membership. Various combinations of linkage methods and distance metrics were used to identify one clustering solution which most correlated with the original distance matrix (i.e., a matrix containing distances between every pair of cases). The possible linkage methods include: Single, Complete, Average Linkage, and the Ward method. Each of these strategies differs in the decision criterion for progressively merging clusters, i.e., based on the distance between the closest points belonging to different clusters (*Single Linkage*), on the furthest apart such points (*Complete Linkage*), on the average distance between points in two clusters (*Average Linkage*), or on which merge would minimize within-cluster variance (*Ward method*, which is similar to *k*-means in that both are least-squares methods; Borcard et al., 2011).

Testing various linkage methods and distance metrics jointly allowed us to find the combination yielding the clustering solution with the highest degree of similarity (i.e., the cophenetic correlation, Jain et al., 1988; Rohlf & Fisher, 1968; Xu & Wunsch, 2009) to the original data. In other words, the solution that has minimal dissimilarity to the raw data.

Due to the relationships between valence, arousal, and dominance displayed in Figure 1 in the main article, distances based on correlations were also used, in addition to Euclidean distances. Each of these was combined with one of the four agglomeration methods, and out of the resulting eight possible combinations, the weakest cophenetic correlation was observed for **Single Linkage** combined with Euclidean distances, *r* = 0.22, whereas in combination with correlation distances, Single Linkage produced a cophenetic correlation of *r* = 0.87. **Complete Linkage**, on the other hand, produced slightly lower correlations with *r* = 0.72 and *r* = 0.77 for Euclidean and correlation distances, respectively. **Ward Linkage** with Euclidean distances (which bears resemblance to *k*-means) produced a cophenetic correlation of *r* = 0.72, and *r* = 0.82 with correlation distances. Finally, **Average Linkage** paired with correlation-based distances produced the most similar results to the original distance matrix, reaching the maximum cophenetic correlation of *r* = 0.91, whereas with Euclidean distances, the correlation dropped to *r* = 0.71. Thus, Average Linkage paired with correlation distances represents the most suitable combination for the IAPS data. The Gower distance (a measure of dissimilarity, Gower, 1971) was also minimized for this same combination: 40030.17, relative to the next best value, achieved by Complete Linkage with correlation distances (190072.7). Single and Ward Linkage both performed more poorly on to this measure.

After having identified the most suitable hierarchical agglomeration method for this dataset, we proceeded to use the clValid R package (Brock et al., 2008) to again determine the most appropriate number of clusters in the data. This package conveniently offers the same combination of agglomeration method and distance metric which was proven to be most suitable for IAPS data.

In terms of both Connectivity and Average Silhouette Widths (briefly described above), a number of two clusters was suggested, whereas the Dunn Index indicated three. Mantel optimality (the correlation between the original distance matrix and binary matrices showing cluster membership; Borcard et al., 2011) also suggested two. However, as with *k*-means, there is some variability to be found, as when using the Elbow method for partitioning variance into clusters (GMD R package; Zhao & Sandelin, 2012), the optimal number of clusters (also based on Average Linkage) indicated was seven.

Finally, using the NbClust package (Charrad et al., 2014) with Average Linkage and correlation distances, and testing a maximum number of fifteen clusters, the most endorsed options (by sixteen indices overall) were two or nine clusters, with fewer other indices suggesting each of: three, four, twelve, or fifteen to extract. When extending the search limit to thirty clusters to extract, two and nine clusters remained the most frequent recommendation (from sixteen indices), with a few other indices suggesting three, four, five, fifteen, twenty-nine, or thirty clusters (the maximum number tested). Thus, similarly to *k*-means, the same tendency is apparent for hierarchical clustering: either a very small, heterogeneous number of clusters is endorsed, or larger numbers of small, fine-grained clusters emerges instead. The notable difference is that, unlike for *k*-means, the nine-cluster (rather than the three-cluster) solution is endorsed much more heavily for hierarchical clustering, as computed with the NbClust package.

**Model-based clustering.** We verified whether the assumptions for model-based clustering were satisfied (i.e., multivariate normality for each component distribution/cluster). To this end, we computed a mixture model using model-based clustering, as implemented within the R package mclust (Fraley & Raftery, 2006). We subsequently examined the eigenvalues and the densities of principal component scores associated with each component in the mixture model. Eigenvalues describe the amount of variation that characterizes each axis in the 3D space where the clusters are defined: ellipsoidal shapes are consistent with normal multivariate distributions, and exhibit larger variations along one axis, and smaller for the other two – as shown in Table S2. Principal component scores largely formed bell-curve shapes for each cluster, suggesting a normal/symmetrical distribution of cases within the 3D space.

Further support for the normality assumption is provided by other multivariate normality tests also listed in Table S2, as well as the 3D density plots for each cluster (or each mixture component), in Figure S1. Thus, we conclude that the conditions of use for model-based clustering were generally satisfied. A variety of model-based clustering models were therefore computed for the data, and their associated BIC values are provided in Table S3. Finally, the BIC-optimal classification of cases (presented alongside uncertainties) is presented in the repository at: [www.github.com/CaterinaC/IAPSClustering2016](file:///C:\Users\s0955633\AppData\Local\Temp\www.github.com\CaterinaC\IAPSClustering2016).

## Validating the clustering solutions

**Finding a stable structure within the data, across methods.** Assuming that the IAPS data presents a clear, discernible structure, all clustering algorithms should in principle be able to identify this structure despite computational differences. In order to check this, we assessed the extent to which model-based clustering yields membership assignments that overlap with those from the other two competing methods.

When using the Adjusted Rand Index (ARI, a conservative measure which penalizes for any randomness in the overlap, Hubert & Arabie, 1985), the degrees of association between the model-based solution and the other two solutions were 0.474 and 0.318, for *k*-means and hierarchical clustering, respectively. The association with an entirely random classification of IAPS cases predictably dropped to ≈ 0. As Steinley (2004) considers ARI values greater than 0.90 - excellent, values greater than 0.80 - good, values greater than 0.65 - moderate, and values less than 0.65 - poor, the values observed for ARI in our dataset seem to indicate each method creates partitions with relatively little in common with the other two.

We next computed Meilă’s (2007) variation of information (VI) criterion (implemented in R package igraph, function compare.communities(); Csardi & Nepusz, 2006), which measures (in bits) how much information is lost or gained by moving from one partitioning to another. This amount should be small if *k* = 5 is an adequate structure for the IAPS data, and is picked up similarly by the various clustering algorithms. The overlap (in terms of VI) between model-based clustering and *k*-means classification is 1.187, and between model-based clustering and hierarchical clustering is 1.462. In order for the overlap to have a possible range between 0 and 1 (where 0 would indicate identical clustering solutions across methods), we normalized values by dividing them with the log of the sample size used (Meilă, 2007). This converted the indices to 0.176 and 0.217, respectively, which can be considered somewhat low values, suggesting there is not much information to be gained/lost when moving from one classification to another, and that there is enough similarity between partitions of five clusters, regardless of the algorithm used to produce them. For details on these measures, please refer to Table S1.

Finally, we used Cramer’s ϕ to assess pairwise overlap between the five-cluster classifications from all three methods. Cross-tabulating *k*-means and model-based classifications led to Cramer’s ϕ = 0.704 (a strong association), whereas crossing model-based and hierarchical classifications led to a lower Cramer’s ϕ = 0.516 (but still indicating a relatively strong association; Kotrlik et al., 2011). Though the ARI results lend some ambiguity, on the whole these results constitute moderate evidence that a specific, five-cluster data structure can be identified in the IAPS, given the level of agreement between the clustering methods.

**Comparing the fit of the model-based clustering solution to that of the other two algorithms.** As a further test, predictive models were built using the interaction term between valence, arousal, and dominance scores, or each of these dimensions on its own, as the outcome, and one of the five-component clustering solutions (*k*-means, hierarchical, and model-based) as the predictor, in order to compare *R*^2^ between them, and thus gauge which clustering method yields groups that most accurately reflect back the original data. This approach resulted in a total of twelve predictive models (four outcomes by three clustering methods). In all cases, models were significant, with *R*^2^ ranging from 0.430 to 0.885, and an average across all methods of *R*^2^ = 0.724 (*SD* = 0.130). This indicates that, on average, 72.4% of the variation seen in outcomes (i.e. raw data) was explained by membership assignment to the five clusters, which is considerable. Strictly referring to model-based clustering, this method achieved *R*^2^ = 0.718 for predicting the interaction term, and *R*^2^ = 0.885 for predicting Valence scores only, *R*^2^ = 0.430 for Arousal and *R*^2^ = 0.744 for Dominance.

In addition, using an additive model building strategy, nested models were compared using the anova() function in R, to verify if each clustering method could explain more variation in the outcomes, above and beyond previously inserted predictors (i.e., clustering solutions). We found that model-based clustering is a complementary method to the other two methods, as each method helped explain significantly more variance in outcomes when added to a model containing one other clustering method. For instance, a model predicting the Valence × Arousal × Dominance interaction based on *k*-means or hierarchical clusters would be improved significantly by the addition of a predictor coding model-based clusters, but also vice versa. Thus we were able to roughly recreate the original data using complementary solutions (i.e., variance explained is not completely overlapping): across all pairwise combinations of predictors, the average *R*^2^ achieved was 0.814 (*SD*=0.016). Specifically, when adding *k*-means or hierarchical clustering classifications to a model including only the model-based classification as a predictor, the boost in *R*^2^ was of 0.11 and 0.08, respectively (significant in both cases at *p* < 0.001). Conversely, when adding the model-based classification as a predictor alongside either of the other two classifications, the rise in *R*^2^ is of 0.02 and 0.20, respectively (*p* < 0.001, in either case).

**Evaluating the stability of the model-based clustering solution.** The IAPS data were divided into two randomly selected halves in order to assess split-half validation. The two halves were arbitrarily defined as *the learning dataset* (used to create a model-based clustering solution with five components), and *the test dataset* (whose clustering solution could be predicted using the solution generated for the learning dataset). If the five-cluster structure found using model-based clustering is appropriate for the IAPS data, then the prediction for the test dataset, and a clustering solution created independently for the test dataset, should match closely.

This procedure was repeated twice, with each random half of the dataset being considered as the learning dataset on a given run. As such, both random halves of the data were subject to model-based clustering, with the specification of extracting five clusters (which was the optimal solution for the dataset considered in its entirety). Subsequently, using the cl_predict() R function from package clue (Hornik, 2005), each classification was used to predict the clustering structure of the other half (the test data).

The degree of association between the predicted clustering of the test dataset and its actual clustering was quantified using a measure of effect size for their cross-tabulation. The average value computed for ϕ Cramer = 0.864, which is a very strong association (Kotrlik et al., 2011). Given this result, the five-cluster solution provided by Mclust() was judged to be robust and well supported by the data.

In addition, in order to reassess how stable the *k* = 5 solution issued by Mclust() was, and/or how much of it was potentially due to multivariate outlying cases, we used a jack-knife procedure to randomly remove 10% of the cases from the dataset, during 7500 bootstrap repetitions^[[3]](#footnote-3)^. Ideally, if the data structure is represented consistently by the clusters, then no major changes should occur with reference to the optimal number of *k*. After each repetition, the optimal value for *k* was reassessed, and across all repetitions, aggregated data suggest the most commonly occurring optimal solution for model-based clustering when 10% of cases were removed was *k* = 3 (46.67%), followed by *k* = 4 (34.67%), and finally, *k* = 5 (18.67%).

Given the departure from the *k* = 5 Mclust() solution achieved on the full dataset, we assessed if there were notable discrepancies between the *k* = 3 and *k* = 5 solutions achieved on the random subsets of the data. Therefore, using 2500 repetitions, on each random data subset we fitted both a three-component and a five-component mixture model using Mclust(), and then assessed the (mis-)match between them. Collectively, the 2500 resulting cross-tabulations achieved an average Cramer’s ϕ = 0.92, with a min = 0.81 and max = 0.99. Therefore the differences seen in the value of *k* most likely reflect the fact that one or two clusters from the *k*=5 solution were collapsed due to the induced data attrition (-10%), but that similarities between solutions nevertheless remained robust.

In order to investigate any further the differences between methods, a similar approach was adopted for *k*-means and hierarchical clustering, by randomly removing 10% of the data during 1000 repetitions. Euclidean distances were used for *k*-means, with correlation distances and Average Linkage being specified for hierarchical clustering. For each of these clustering methods and during each repetition, the adequate number for *k* was recomputed based on measures of Connectivity, Average Silhouette Width, and Dunn Index, offered by the clValid() R function (within package clValid by Brock et al., 2008). After 10% of the values had been removed, 82.80% and 71.63% of the time, only one cluster emerged for *k*-means and hierarchical clustering, respectively (i.e., no cluster structure could be defined). Given that the variability in the dataset leads to ≈ 60% of data points having Mahalanobis distances larger than 2 (and peaking at 19.559), extracting one single cluster was judged to be an unrealistic solution, and symptomatic of an inefficient clustering process for this particular dataset. This suggests that model-based clustering is more appropriate for IAPS data, relative to *k*-means or hierarchical clustering.

# References

Ball, G. H. & Hall, D. J. (1965). *ISODATA, a novel method of data analysis and pattern classification* (Technical report). Menlo Park, CA: Stanford Research Institute.

Berry, K. J., & Mielke Jr, P. W. (1992). Indices of ordinal variation. *Perceptual and Motor Skills, 74*(2), 576-578.

Blair, J., & Lacy, M. G. (2000). Statistics of ordinal variation. *Sociological Methods and Research, 28*(3), 251-280.

Borcard, D., Gillet, F., & Legendre, P. (2011). *Numerical ecology with R*. New York: Springer

Brock, G., Pihur, V., Datta, S., & Datta, S. (2008). clValid: An R package for cluster validation. *Journal of Statistical Software, 25*(4), 1-22. Retrieved from: http://www.jstatsoft.org/v25/i04/paper.pdf, on August 14, 2013.

Brown, C. (1998). *Applied multivariate statistics in geohydrology and related sciences.* Berlin: Springer Science & Business Media.

Caliński, T. & Harabasz, J. (1974). A dendrite method for cluster analysis*. Communications in Statistics - Theory and Methods, 3*(1), 1–27.

Charrad, M., Ghazzali, N., Boiteau, V., & Niknafs, A. (2014). NbClust: an R package for determining the relevant number of clusters in a data set. *Journal of Statistical Software*, *61*(6), 1-36.

Csardi, G. & Nepusz, T. (2006). The igraph software package for complex network research. *InterJournal Complex Systems, 1695*(5), 1–9.

Dimitriadou, E., Dolničar, S., & Weingessel, A. (2002). An examination of indexes for determining the number of clusters in binary data sets. *Psychometrika, 67*(1), 137–159.

Dimitriadou, E. (2014). *cclust: Convex Clustering Methods & Clustering Indexes* (version 0.6-19) [R package and documentation]. Retrieved from: http://CRAN.R-project.org/package=cclust, on June 20, 2015

Dolnicar, S., Grabler, K., Mazanec, J. A. (1999). A tale of three cities: Perceptual charting for analysing destination images. In A. G. Woodside, G. I. Crouch, J. A. Mazanec, M. Opperman, & M. Y. Sakai (Eds.), *Consumer psychology of tourism, hospitality and leisure* (pp. 39-62)*.* Wallingford, UK: CAB International Publishing.

Dunn, J. C. (1973). A fuzzy relative of the ISODATA process and its use in detecting compact well-separated clusters. *Journal of Cybernetics, 3*(3), 32–57.

Fraley, C., & Raftery, A. E. (2006). *mclust version 3: An R package for normal mixture modeling and model-based clustering* (Report No. 504). Seattle, WA: University of Washington, Dept. of Statistics.

C. Fraley, A. E. Raftery, T. B. Murphy and L. Scrucca (2012). *mclust version 4 for R: Normal Mixture Modeling for Model-Based Clustering, Classification, and Density Estimation*. (Report No. 597). Seattle, WA: University of Washington, Dept. of Statistics.

Gower, J. C. (1971). A general coefficient of similarity and some of its properties. *Biometrics, 27*(4), 857-871.

Handl, J., Knowles, J., & Kell, D. B. (2005). Computational cluster validation in post-genomic data analysis. *Bioinformatics, 21*(15), 3201–3212.

Hartigan, J. A. (1975). *Clustering algorithms*. New York: Wiley.

Hornik, K. (2005). A CLUE for CLUster ensembles. *Journal of Statistical Software*, *14*(12), 1-25.

Hubert, L. & Arabie, P. (1985). Comparing partitions. *Journal of Classification, 2*(1), 193–218.

Jain, A. K., & Dubes, R. C. (1988). *Algorithms for clustering data* (Vol. 6). Englewood Cliffs: Prentice Hall.

Jarek, S. (2009). *Mvnormtest: Normality test for multivariate variables (version 0.1-9)* [R package and documentation]. Retrieved from: http://cran.r-project.org/web/packages/mvnormtest/index.html, on January 12, 2016

Korkmaz S., Goksuluk D., Zararsiz G. (2014). MVN: An R Package for Assessing Multivariate Normality. *The R Journal, 6*(2):151-162.

Kotrlik, J. W., Williams, H. A., & Jabor, M. K. (2011). Reporting and interpreting effect size in quantitative agricultural education research. *Journal of Agricultural Education, 52*(1), 132–142.

Kvalseth, T. O. (1995). Coefficients of variation for nominal and ordinal categorical data. *Perceptual and Motor Skills, 80*(3), 843-847.

Lucas, A. (2014). *amap: Another Multidimensional Analysis Package (version 0.8-14)* [R package and documentation]. Retrieved from: *http://CRAN.R-project.org/package=amap*, on January 12, 2016

Mazanec, J. A., & Strasser, H. (2000). *A nonparametric approach to perceptions-based market segmentation: Foundations* (Vol. 1). New York: Springer.

Meilă, M. (2007). Comparing clusterings - An information based distance. *Journal of Multivariate Analysis, 98*(5), 873–895.

Preston, C. C., & Colman, A. M. (2000). Optimal number of response categories in rating scales: reliability, validity, discriminating power, and respondent preferences. *Acta psychologica, 104*(1), 1-15.

Rohlf, F. J. & Fisher, D. R. (1968). Tests for hierarchical structure in random data sets. *Systematic Biology, 17*(4), 407–412.

Rousseeuw, P. J. (1987). Silhouettes: a graphical aid to the interpretation and validation of cluster analysis. *Journal of Computational and Applied Mathematics, 20*(1), 53–65.

Steinley, D. (2004). Properties of the Hubert-Arabie Adjusted Rand Index. *Psychological Methods*, *9*(3), 386.

Xu, R. & Wunsch, D. (2009). *Clustering* (Vol. 10). New Jersey: John Wiley & Sons.

Zhao, X. & Sandelin, A. (2012). GMD: Measuring the distance between histograms with applications on high-throughput sequencing reads. *Bioinformatics, 28*(8), 1164–1165.

# Tables

Table S1. Principal clustering indices consulted.

| Index name | Formula | Abbreviations | Eq. |
| --- | --- | --- | --- |
| Calinski-Harabasz | $\frac{SSB/(k-1)}{SSW/(N-k)}$ | *SSB* = Sum of squares between;  *SSW* = Sum of squares within;  *k* = number of clusters;  *N* = number of data points. | **(1)** |
| Ball | $\frac{ssw}{k}$ |  | **(2)** |
| Hartigan | $\log\left( \frac{SSB}{SSW} \right)$ |  | **(3)** |
| SSI | $SSI=\sum_{j=1}^{M} \left( \mathcal{w}_{i}{max}_{j}- \mathcal{w}_{i}{min}_{j} \right)$  $\mathcal{w}SSI= \frac{SSI}{{SSI}_{max}}\frac{N_{max \vee min}}{N}$, where $0< \mathcal{w}SSI<1$ |  | **(4)** |
| Dunn | $\frac{\min_{C_{k}, C_{l}\mathcal{\in C,}C_{k}\neq C_{l}} \left( \min_{i\in C_{k}, j\in C_{l}} dist\left( i, j \right) \right)}{\max_{C_{m}\mathcal{\in C}} diam\left( C_{m} \right)}$ | 𝒞 = a particular clustering partition;  $diam\left( C_{m} \right)$ = maximum distance between observations in cluster $C_{m}$. | **(5)** |
| Connectivity | $\sum_{i=1}^{N} \sum_{j=1}^{L} x_{{i,nn}_{i(j)}}$ | *L* = parameter giving the nearest neighbors to use;  ${nn}_{i(j)}$ = *j*^th^ nearest neighbor of observation *i;*  $x_{{i,nn}_{i(j)}}$ = 0, if *i* and *j* are in the same cluster, and 1/*j* otherwise. | **(6)** |
| ASW | $\bar{\frac{b_{i}-a_{i}}{\max\left( b_{i}, a_{i} \right)}}$ | $a_{i}$ = the average distance between *i* and all other observations in the same cluster;  $b_{i}$ = the average distance between *i* and the observations in the nearest neighboring cluster. | **(7)** |
| ARI | $\frac{\sum_{i,j} \left( \begin{matrix} n_{ij} \\ 2 \end{matrix} \right)-\left[ \sum_{i} \left( \begin{matrix} a_{i} \\ 2 \end{matrix} \right)\sum_{j} \left( \begin{matrix} b_{j} \\ 2 \end{matrix} \right) \right]/\left( \begin{matrix} n \\ 2 \end{matrix} \right)}{\frac{1}{2}\left[ \sum_{i} \left( \begin{matrix} a_{i} \\ 2 \end{matrix} \right)+\sum_{j} \left( \begin{matrix} b_{j} \\ 2 \end{matrix} \right) \right]-\left[ \sum_{i} \left( \begin{matrix} a_{i} \\ 2 \end{matrix} \right)\sum_{j} \left( \begin{matrix} b_{j} \\ 2 \end{matrix} \right) \right]/\left( \begin{matrix} n \\ 2 \end{matrix} \right)}$ | Formula equivalent to:  $AdjustedRandIndex=\frac{RandIndex-ExpectedIndex}{MaximumIndex-ExpectedIndex}$ , where:  $\left( \begin{matrix} n \\ 2 \end{matrix} \right)=$ number of pairs of observations;  $\left( \begin{matrix} a_{i} \\ 2 \end{matrix} \right)=$the number of distinct pairs that can be constructed within rows;  $\left( \begin{matrix} b_{j} \\ 2 \end{matrix} \right)=$ the number of distinct pairs that can be constructed within columns. | **(8)** |
| VI | $\mathcal{H}\left( \mathcal{C} \right)+ \mathcal{H}\left( \mathcal{C'} \right)-2I\left( \mathcal{C,C'} \right)$ | $I\left( \mathcal{C,C'} \right)=$ mutual information between two clusterings;  $\mathcal{H}\left( \mathcal{C} \right)$ = the entropy associated with clustering $\mathcal{C}$. | **(9)** |

*Note*. For **(1)**, **(2)**, **(3)**, see e.g., Dimitriadou, Dolničar & Weingessel (2002). For **(4)**, see Mazanec & Strasser (2000). For **(5)**, **(7)**, **(6)**, see Brock et al. (2008). For **(8)**, see Hubert & Arabie (1985). For **(9)**, see Meila (2007).

Table S2. Results from various multivariate normality tests run in R, using packages MVN (Korkmaz et al., 2014), mvnormtest (Jarek, 2009) and amap (Lucas, 2014). No cluster is shown to be non-normal by all of the tests simultaneously.

| Test | Measure & Value | | Cluster 1 | Cluster 2 | Cluster 3 | Cluster 4 | Cluster 5 |
| --- | --- | --- | --- | --- | --- | --- | --- |
| Mardia | Skewness | χ2 (*p*) | 12.43 (0.26) | *27.09 (0.00)* | *22.1 (0.02)* | 15.44 (0.12) | 14.01 (0.17) |
|  | Kurtosis | z (*p*) | -1.53 (0.13) | 1.05 (0.29) | -0.06 (0.96) | -1.34 (0.18) | *-2.92 (0.00)* |
| Shapiro-Wilk | | W (*p*) | 0.99 (0.08) | *0.96 (0.02)* | *0.95 (0.01)* | *0.98 (0.03)* | 0.992 (0.08) |
| Henze-Zirkler | | Hz (*p*) | *1.18 (0.01)* | 0.93 (0.07) | *1.03 (0.02)* | *1.12 (0.02)* | 0.98 (0.11) |
| Royston | | H (*p*) | 3.88 (0.19) | 4.67 (0.17) | *14.51 (0.00)* | 5.67 (0.12) | 5.45 (0.14) |
| PCA | Eigenvalue 1 | | 21.57 | 11.77 | 11.03 | 15.04 | 21.20 |
|  | Eigenvalue 2 | | 14.44 | 7.91 | 8.05 | 14.05 | 19.94 |
|  | Eigenvalue 3 | | 7.43 | 3.00 | 4.86 | 5.43 | 9.13 |

*Note:* Significant results indicating non-normality are printed using italic characters. Wherever rounding has resulted in near-zero values, these should be interpreted as *p*<0.001.

Table S3. BIC values for all the models considered within the model-based clustering procedure.

| *k* | EII | VII | EEI | VEI | EVI | VVI | EEE | EVE | VEE | VVE | EEV | VEV | EVV | VVV |
| --- | --- | --- | --- | --- | --- | --- | --- | --- | --- | --- | --- | --- | --- | --- |
| 1 | -8671.93 | -8671.93 | -8429.92 | -8429.92 | -8429.92 | -8429.92 | -6853.79 | -6853.79 | -6853.79 | -6853.79 | -6853.79 | -6853.79 | -6853.79 | -6853.79 |
| 2 | -7550.51 | -7484.72 | -7198.02 | -7174.51 | -7167.27 | -7151.06 | -6589.67 | -6449.51 | -6574.74 | -6446.17 | -6438.96 | -6442.29 | -6445.84 | -6448.33 |
| 3 | -7074.81 | -7072.43 | -6933.30 | -6929.80 | -6913.77 | -6913.51 | -6421.70 | -6404.12 | -6408.78 | -6403.04 | -6385.87 | -6351.59 | -6390.38 | -6368.51 |
| 4 | -6933.33 | -6895.56 | -6759.19 | -6711.12 | -6745.23 | -6713.03 | -6418.85 | -6410.76 | -6360.55 | -6386.42 | -6394.85 | -6343.72 | -6404.55 | -6383.36 |
| 5 | -6791.55 | -6805.93 | -6644.96 | -6660.17 | -6663.38 | -6686.11 | -6382.17 | -6376.99 | -6347.73 | -6380.00 | -6374.04 | **-6341.11** | -6388.91 | -6378.29 |
| 6 | -6747.01 | -6760.70 | -6636.97 | -6653.35 | -6669.52 | -6663.25 | -6399.60 | -6400.29 | -6373.22 | -6395.85 | -6401.69 | -6378.84 | -6443.89 | -6408.86 |
| 7 | -6733.89 | -6745.28 | -6636.13 | -6661.72 | -6679.37 | -6662.53 | -6407.49 | -6428.39 | -6377.73 | -6439.33 | -6442.38 | -6417.70 | -6491.33 | -6498.67 |
| 8 | -6726.10 | -6695.73 | -6594.54 | -6611.93 | -6638.20 | -6665.66 | -6433.14 | -6449.76 | -6408.65 | -6451.17 | -6460.30 | -6446.90 | -6494.17 | -6514.12 |
| 9 | -6645.08 | -6664.46 | -6594.39 | -6601.02 | -6637.80 | -6650.03 | -6421.71 | -6451.09 | -6411.01 | -6471.65 | -6461.81 | -6491.22 | -6554.31 | -6559.11 |

*Note:* According to Fraley et al. (2012), the abbreviations refer to the configuration of the clusters in a given model: EII = Spherical distribution, equal volume, equal shape, orientation not applicable; VII = Spherical distribution, variable volume, equal shape, orientation not applicable; EEI = Diagonal distribution, equal volume, equal shape, orientation on coordinate axes; VEI = Diagonal distribution, variable volume, equal shape, orientation on coordinate axes; EVI = Diagonal distribution, equal volume, variable shape, orientation on coordinate axes; VVI = Diagonal distribution, variable volume, variable shape, orientation on coordinate axes; EEE = Ellipsoidal distribution, equal volume, equal shape, equal orientation; EEV = Ellipsoidal distribution, equal volume, equal shape, variable orientation; VEV = Ellipsoidal distribution, variable volume, equal shape, variable orientation; VVV = Ellipsoidal distribution, variable volume, variable shape, variable orientation. Here, the BIC value underlined and in bold signals the optimal model, with *k*=5, and VEV as the most suitable configuration.

# Figures


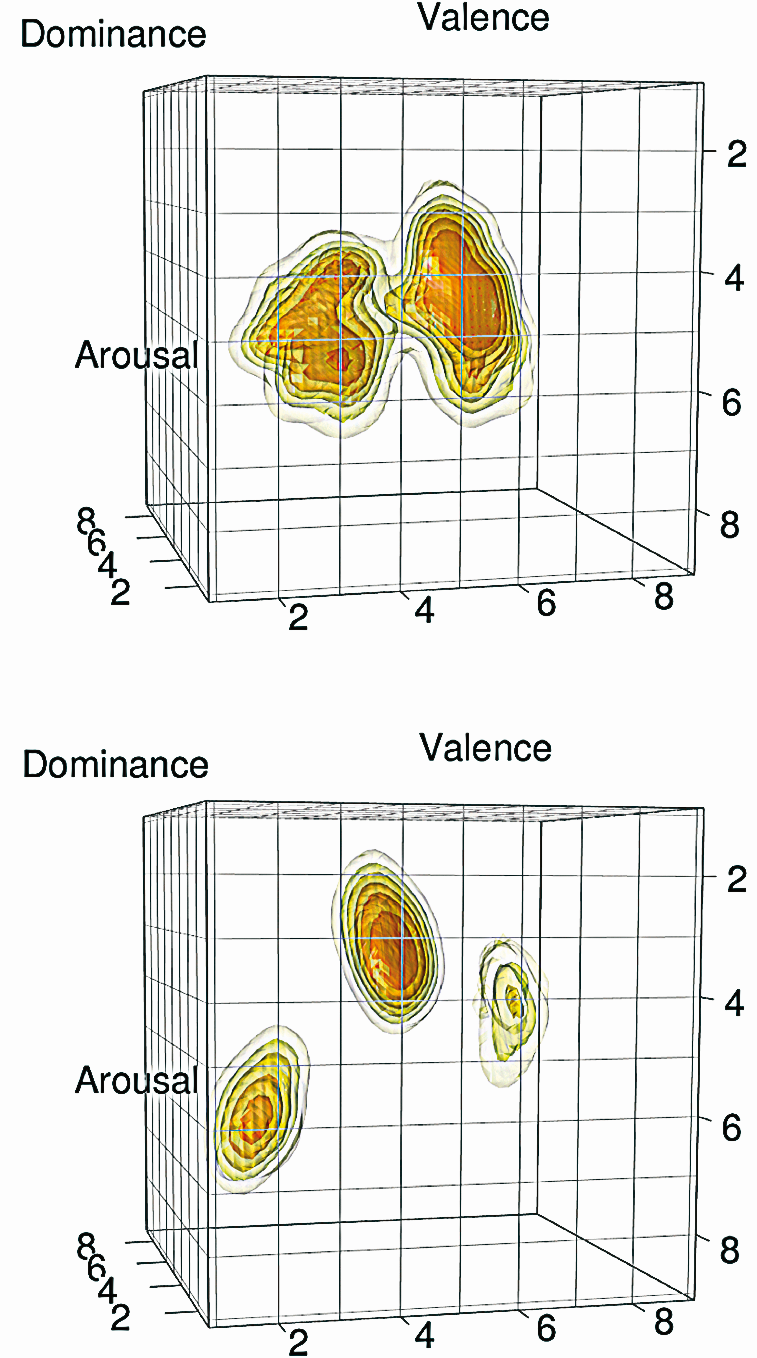


Figure S1. Density according to cluster, largely confirming the VEV model issued by Mclust(): varying volume/equal shape (ellipsoidal)/varying orientation. In the top section: clusters 1 and 5, in the lower section: clusters 2, 3 and 4.

1. *cv* = (*sd* / *m*) × 100. The *cv* usually requires a *ratio* scale with an absolute 0 value, to allow for scale transformations. While the nine-point PAD scales can be seen as *interval* scales with adequate measurement properties (Preston & Colman, 2000), they lack an absolute 0. Regardless, because in practice the IAPS scales are not converted to any other system of measurement, using the *cv* on IAPS data was considered an acceptable step. [↑](#footnote-ref-1)
2. Although other options exist for computing distances between data points, Euclidean distances are the most common for *k*-means, particularly as this clustering method was developed with them in mind (Hartigan & Wong, 1979; Jain, 2010). [↑](#footnote-ref-2)
3. Due to limitations in computing power, this number will vary across the various procedures used to validate the clustering solution. [↑](#footnote-ref-3)
